# What’s new in bone forming tumours of the skeleton?

**DOI:** 10.1007/s00428-019-02683-w

**Published:** 2019-11-18

**Authors:** Natasja Franceschini, Suk Wai Lam, Anne-Marie Cleton-Jansen, Judith V. M. G. Bovée

**Affiliations:** Department of Pathology, Leiden University Medical Center, P.O. Box 9600, L1-Q, 2300 RC Leiden, Netherlands

**Keywords:** Osteoid osteoma, Osteoblastoma, Osteosarcoma, Molecular pathology, FOS

## Abstract

Bone tumours are difficult to diagnose and treat, as they are rare and over 60 different subtypes are recognised. The emergence of next-generation sequencing has partly elucidated the molecular mechanisms behind these tumours, including the group of bone forming tumours (osteoma, osteoid osteoma, osteoblastoma and osteosarcoma). Increased knowledge on the molecular mechanism could help to identify novel diagnostic markers and/or treatment options. Osteoid osteoma and osteoblastoma are bone forming tumours without malignant potential that have overlapping morphology. They were recently shown to carry *FOS* and—to a lesser extent—*FOSB* rearrangements suggesting that these tumours are closely related. The presence of these rearrangements could help discriminate these entities from other lesions with woven bone deposition. Osteosarcoma is a malignant bone forming tumour for which different histological subtypes are recognised. High-grade osteosarcoma is the prototype of a complex karyotype tumour, and extensive research exploring its molecular background has identified phenomena like chromothripsis and kataegis and some recurrent alterations. Due to lack of specificity, this has not led to a valuable novel diagnostic marker so far. Nevertheless, these studies have also pointed towards potential targetable drivers of which the therapeutic merit remains to be further explored.

## Introduction

Bone tumours are rare and therefore considered difficult to diagnose and treat. They comprise a heterogeneous group of tumours, where most subtypes have a distinct clinical and histological presentation.

Histologically, over 60 different bone tumours are recognised. Some are difficult to separate as there can be extensive morphological and even immunohistochemical overlap. Distinction is important as these tumours differ in clinical behaviour and thus in required treatment. In recent years, many papers have been published unravelling the molecular background of several bone tumours, mostly using deep sequencing techniques. From the molecular point of view, these tumours can be roughly divided in two main groups, as a conceptual framework [1]: tumours can either have a simple or complex karyotype. The group of tumours with a simple karyotype are usually monomorphic and driven by a specific mutation or translocation. The tumours with complex karyotype are more often pleomorphic, show aneuploidy, with many copy number alterations and (random) translocations and mutations.

The group of skeletal tumours that are characterised by bone deposition contains osteoma, osteoid osteoma, osteoblastoma and osteosarcoma (Table 1). Osteoma is benign and composed of mature lamellar bone, has a simple karyotype and occurs more often in patients with Gardner’s syndrome, that harbour a germline mutation in the *APC* gene. Osteoid osteoma and osteoblastoma are histologically identical, have a simple karyotype and deep sequencing studies have recently unravelled a recurrent translocation [2]. This is in contrast with high-grade osteosarcoma, for which a complex karyotype showing aneuploidy, multiple copy number alterations, (random) translocations and mutations is the hallmark [3]. This review will focus on osteoid osteoma/osteoblastoma and high-grade osteosarcoma, as examples for simple karyotype, translocation driven versus complex karyotype tumours, respectively.

**Table 1:** Clinical features, radiology, karyotype and molecular pathology of osteoma, osteoid osteoma, osteoblastoma and conventional osteosarcoma

	Osteoma	Osteoid osteoma	Osteoblastoma	Conventional osteosarcoma
Clinical features	• Benign	• Benign	• Locally aggressive	• Malignant
	• Mostly found incidentally	• < 2 cm in size	• > 2 cm in size	• Located at metaphysis of long bones
	• Located at bone surface	• Located in long bones	• Located in posterior column of spine	
Radiology	Homogenous and sharply demarcated tumour	Oval radiolucency (nidus) with surrounding sclerosis	Often lytic lesion , may be alike aneurysmal bone cyst	Lytic, sclerotic or mixed lesion, often expanding into surrounding soft tissue
Karyotype	Simple karyotype	Simple karyotype	Simple karyotype	Complex karyotype
Molecular pathology	Can be associated with Gardner’s syndrome: germline *APC* mutation	*FOS* and to a lesser extent *FOSB* translocations	*FOS* and to a lesser extent *FOSB* translocations	Chromothripsis and kateagis with most often alterations in *TP53*

## Osteoid osteoma and osteoblastoma

Novel *FOS* and *FOSB* rearrangements were recently found in osteoid osteoma and osteoblastoma [2]. These tumours account for 3% and 1% of all primary bone tumours, respectively [4]. These two entities are histologically similar and only slightly differ in their clinical presentation. At present, they are arbitrarily divided by tumour size below or above 2 cm in diameter, although the recent finding show that they share the same molecular alteration might suggest that they represent the same disease [4–6].

### Clinical presentation

Osteoid osteoma and osteoblastoma typically present during the second decade of life, with men being overrepresented (male to female ratio 2:1) [4]. Osteoid osteoma is usually located at the long bones in the lower extremity, but other commonly described sites involve the spine, upper extremity, hands, feet and pelvis [4, 5, 7]. The most prominent clinical symptom of osteoid osteoma is frequent and severe night pain that responds adequately to nonsteroidal anti-inflammatory drugs (NSAIDs) [4, 5]. Osteoblastoma is larger in size, and the majority is localized in the posterior column of the spine [4, 5, 8], resulting in neurologic symptoms as a recurring sign [4]. Pain is frequently present, but in contrast to osteoid osteoma, it does not respond to administration of NSAIDs [4, 5]. Both osteoid osteoma and osteoblastomas have no malignant potential, although osteoblastoma can behave as a locally aggressive tumour [4]. For radiologists, the diagnosis of osteoid osteoma is usually straight forward, showing a characteristic oval radiolucency (nidus) with surrounding sclerosis, while osteoblastoma can be accompanied by a more broad differential diagnosis depending on its location, including aneurysmal bone cyst, giant cell tumour of bone and osteosarcoma [4, 9].

### Histology

**Fig. 1:**
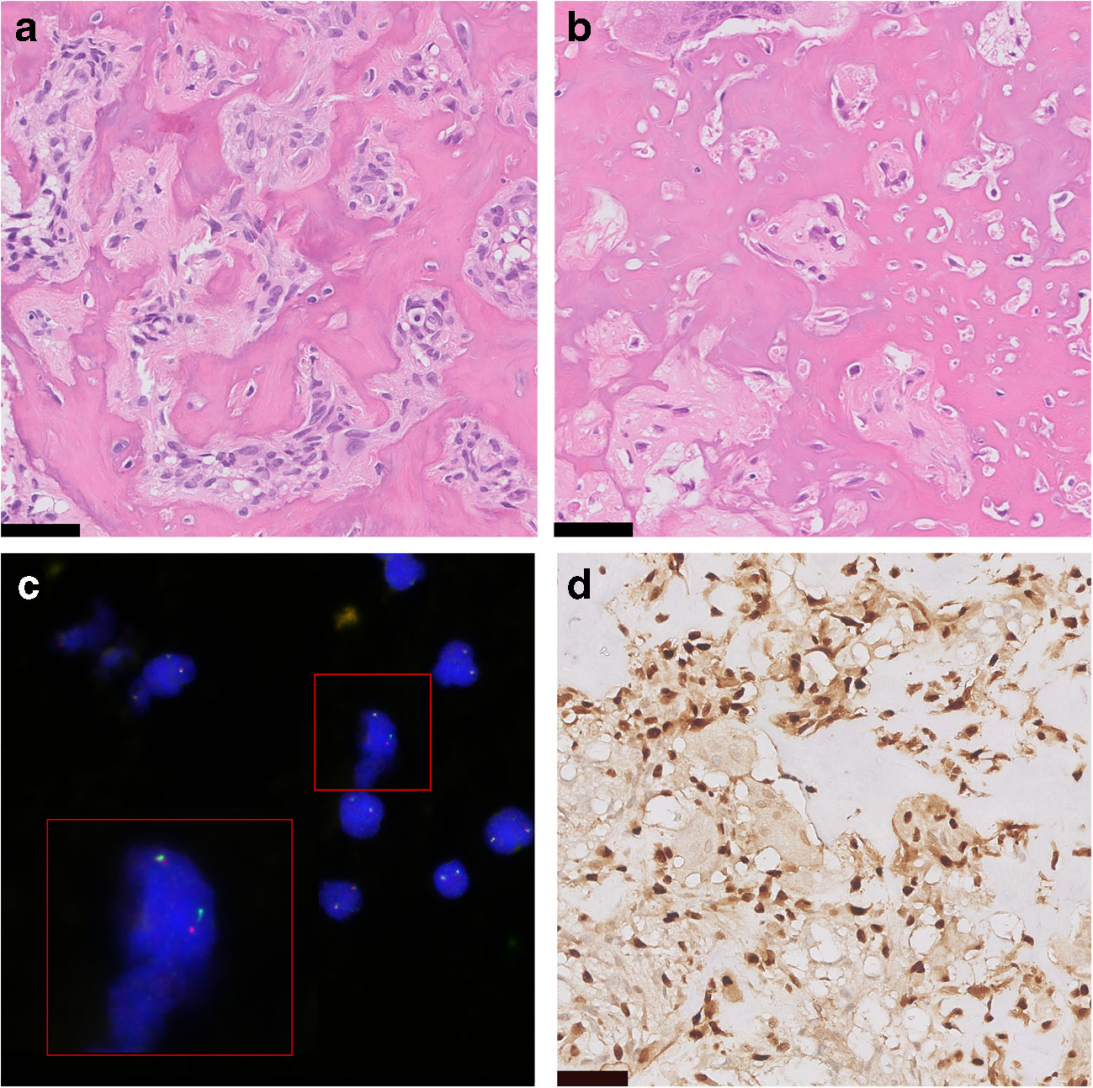
Osteoid osteoma and osteoblastoma. **a** Osteoid osteoma. **b** Osteoblastoma show identical morphology at haematoxylin and eosin staining, with deposition of woven bone by osteoblast-like tumour cells. **c** Fluorescence in situ hybridization (FISH) showing *FOS* rearrangement in osteoblastoma. **d** Immunohistochemical staining for FOS in osteoblastoma showing nuclear overexpression in the tumour cells. Scale bar is 50 μm

Osteoid osteoma and osteoblastoma are histologically indistinguishable [10] (Fig. 1a, b). Both tumours are composed of irregular trabeculae of woven bone, lined with active osteoblasts. In osteoid osteoma, the central area of the lesion (nidus) is sharply demarcated and surrounded by hyper-vascularized sclerotic bone. In between the trabeculae, there is loose vascularised stroma, and small osteoclast-like giant cells are frequently seen [7, 11]. Osteoblastoma can show slightly more haphazardly arranged trabeculae [6]. Additional aneurysmal bone cyst (ABC)-like changes can be present, especially in larger tumours [4]. The term epithelioid osteoblastoma is reserved for osteoblastomas with the presence of large osteoblasts with an epithelioid appearance. Surrounding cytoplasm is abundant, and nuclei are hyperchromatic or show prominent nucleoli [4]. The most important differential diagnosis includes osteoblastoma-like osteosarcoma, that is distinguished from osteoblastoma based on the presence of host-bone infiltration and lack of differentiation towards the periphery [12]. However, this can be difficult to appreciate in small biopsies or curettage specimens. Definitive diagnosis is always made based on radiological and clinicopathological correlation.

### Molecular pathology

Before the elucidation of the genetic background of osteoid osteoma and osteoblastoma, clonal chromosome aberrations were reported in two osteoblastomas, with structural alterations involving 22q13.1 [13], and only non-recurrent rearrangements were found using cytogenetic studies [14]. In 2018, in a quiet genomic background with paucity of somatic alterations, recurrent *FOS* and—to a lesser extent—*FOSB* rearrangements were found in both osteoid osteoma and osteoblastoma using RNA sequencing, demonstrating that both tumours were similar at the molecular level. In 5 out of 6 cases, *FOS* rearrangements were present, while the remaining case showed rearrangements involving its paralogue, *FOSB*. All *FOS* breakpoints were exonic and involved exon 4. Rearrangement partners were both introns of others genes (*ANKH, KIAA1199, MYO1B*) or intergenic regions [2]. Equivalent to *FOS* rearranged epithelioid hemangioma [15, 16], stop codons were encountered at, or early after the break points, leading to truncation of the protein with retention of the leucine zipper, and therefore its function as a transcription factor. Functional studies in epithelioid hemangioma demonstrated that the truncated protein was more resistant to degradation [17]. In the *FOSB* rearranged osteoblastoma, rearrangement resulted in an in frame fusion connecting *PPP1R10* to *FOSB*, leading to altered signalling, due to promotor swapping [2]. Strikingly, *FOSB* fusions were also involved in pseudomyogenic hemangioendothelioma and atypical epithelioid hemangioma, resulting in promoter swapping [18, 19]. As genetic alterations in these vascular tumours are identical to those found in osteoid osteoma and osteoblastoma, one can speculate that a comparable molecular mechanism of tumorigenesis is operable in osteoid osteoma and osteoblastoma.

These novel molecular findings have provided new tools to improve diagnostic accuracy, as both fluorescence in situ hybridization (FISH) and immunohistochemical staining can detect *FOS* rearrangements (Fig. 1c, d). FISH was performed in an independent cohort and showed in the majority of cases rearrangements involving *FOS* and to a lesser extent *FOSB* [2]. In a follow-up study, immunohistochemistry showed strong and diffuse nuclear staining in the majority (79%) of osteoid osteomas and osteoblastomas, using a FOS antibody against the N terminus [20]. However, a previously published small study cohort demonstrated that osteoid osteoma and osteoblastoma lacked strong nuclear expression of FOS, indicating variability in sensitivity between different antibodies [21]. In terms of specificity, strong nuclear expression of FOS has been detected in a subset of other bone forming tumours and was only rarely present in osteosarcoma [2, 21]. Notably, in mouse models, the *c-fos* oncogene caused osteosarcoma, when fused with a highly active promotor and the *v-fos* 3’ untranslated region [22]. This is intriguing as in human tumours *FOS* and *FOSB* rearrangements have so far only been identified in vascular and bone forming tumours lacking malignant potential [15, 16, 18, 19].

## Osteosarcoma

Osteosarcoma is the most common primary malignant tumour of the bone [23]. The 5-year overall survival for osteosarcoma patients is 71% and has not improved in the last decades, clearly indicating that novel therapeutic strategies are needed [24]. Fortunately, many papers have been published gradually unravelling the pathogenesis of osteosarcoma, which might help develop new therapeutic targets.

### Clinical presentation

Primary high-grade osteosarcoma occurs most often in young children and adolescents, but there is a second peak at a later age. In the latter group, osteosarcoma can occur secondary to radiation or Paget’s disease [25]. Osteosarcoma has a slight male predominance [26]. Patients with osteosarcoma often show signs of localised deep pain, especially manifest at night, developed over a longer period of a few weeks to months. This could also be in combination with limited mobility or localised warmth. A palpable mass can be present, which is tender during physical examination [27].

For diagnosis of conventional osteosarcoma, a radiograph is made in two planes, in which the lesion appears as lytic, sclerotic or mixed lytic and sclerotic. This lesion often expands into the surrounding soft tissue, with periosteal reaction and destruction of cortical bone [28]. MRI or CT imaging may provide additional information, guiding the subsequent biopsy of the lesion [28].

### Histology

**Fig. 2:**
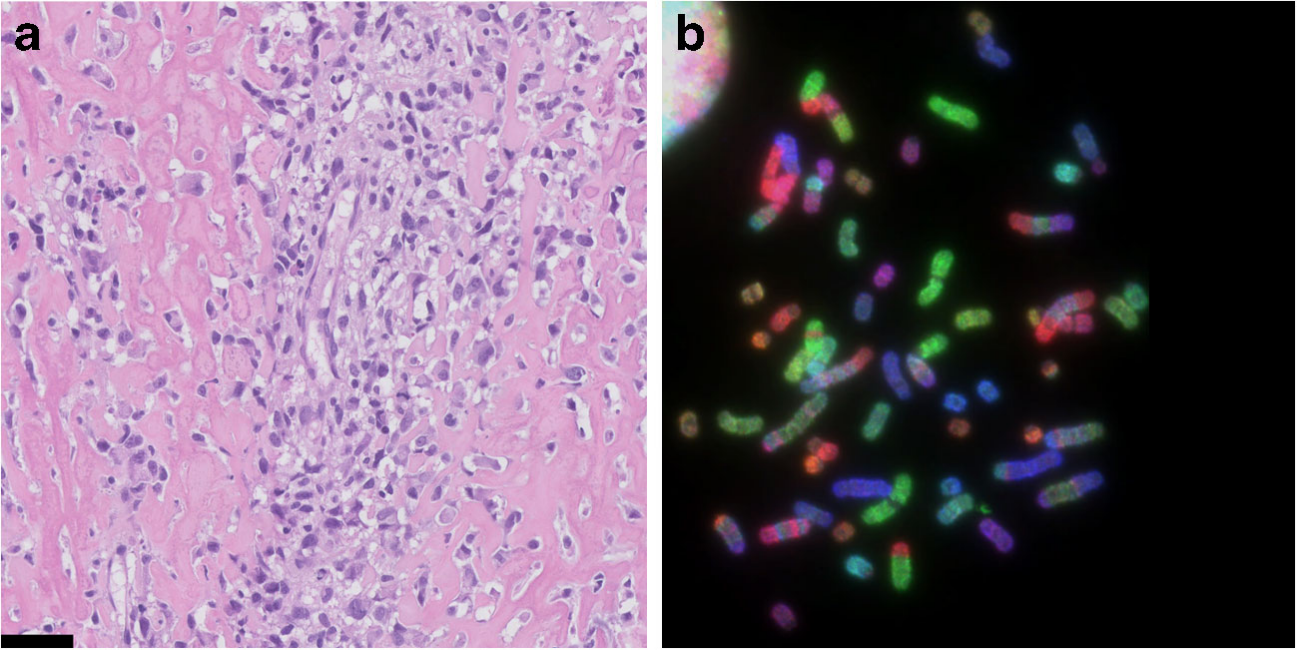
High-grade osteosarcoma. **a** Conventional osteoblastic osteosarcoma showing atypical cells with abundant deposition of osteoid (haematoxylin and eosin staining). Scale bar is 50 μm. **b** Combined binary ratio fluorescence in situ hybridization (COBRA-FISH) showing complex numerical and structural changes which is characteristic of high-grade osteosarcoma

The presence of osteoid, the unmineralized extracellular matrix produced by the tumour cells, is the hallmark of osteosarcoma and visible as a pink dense structure in haematoxylin and eosin stained sections (Fig. 2a). Mineralization can occur. Osteosarcoma can arise in the medulla (central) or at the bone surface. Different osteosarcoma subtypes are recognised, based on their clinical presentation in combination with histological and molecular features (Table 2) [26]. High grade central osteosarcoma is the most common subtype, and most papers published over the last decade, as well as this review, focus on this subtype.

**Table 2:** Osteosarcoma subtypes

Subtype	Location	Grade	Histology
Low-grade central osteosarcoma	Medulla	Low grade	Spindle cells with low-grade nuclear atypia and well-formed neoplastic woven bone trabeculae, often 12q13 amplification
Parosteal osteosarcoma	Surface	Low grade	Spindle cell proliferation, often with cartilaginous differentiation, and 12q13 amplification
Periosteal osteosarcoma	Surface (typically underneath the periosteum)	Intermediate grade	Predominantly chondroblastic bone-forming sarcoma
Conventional osteosarcoma	Medulla	High grade	High-grade sarcoma in which the tumour cells produce bone. Tumour cells can be fibroblastic, chondroblast- or osteoblast-like
Fibroblastic Chondroblastic Osteoblastic			
Small-cell osteosarcoma	Medulla	High grade	Small cells with scant cytoplasm, associated with variable osteoid formation; may resemble Ewing sarcoma
Telangiectatic osteosarcoma	Medulla	High grade	Osteosarcoma composed of blood-filled or empty cystic spaces closely simulating aneurysmal bone cyst
High-grade surface osteosarcoma	Surface	High grade	Similar to conventional osteosarcoma

### Germline predisposition to osteosarcoma

Certain hereditary syndromes predispose to osteosarcoma, such as Li-Fraumeni syndrome (mutations in *TP53* or, less frequently, *CHEK2*), retinoblastoma (mutations in *RB1*) and Rothmund-Thomson syndrome (mutations in *RECQL4*) [29–31]. Other hereditary syndromes with germline mutations in RecQ-like helicases, including RAPADILINO syndrome, Baller-Gerold syndrome, Werner syndrome and Bloom syndrome, also have an increased risk for osteosarcoma [32]. Another hereditary syndrome in which a helicase is mutated is ATR-X syndrome (alpha-thalassemia mental retardation syndrome). Patients with ATR-X syndrome show intellectual disability and skeletal abnormalities. Recently, two patients have been reported with ATR-X syndrome that developed osteosarcoma [33, 34].

### Molecular alterations in osteosarcoma

High-grade osteosarcoma is characterised by a complex karyotype with many amplifications, deletions and (random) translocations (Fig. 2b). This complex genome hampers identification of the driver genes causing genome instability: very few recurrent alterations have been identified in osteosarcoma. One mechanism explaining the genomic instability in osteosarcoma is chromothripsis, the shattering of one or a few chromosomes into small fragments that are stitched together in a random order and orientation [35]. It was first discovered by Stephens et al. in chronic lymphocytic leukaemia, chordoma and osteosarcoma [35]: chromothripsis occurs in 3% of all cancers and in 30% of osteosarcomas. A more recent study confirmed chromothripsis in osteosarcoma, but showed a higher percentage—nearly 90%—where tumours with chromothripsis also frequently harbour amplifications [36]. The discrepancy may be attributed to the uncertain definition of chromothripsis. Exome sequencing shows a relatively low mutational burden in osteosarcoma ranging from 0.3–1.2 mutations per mega base; however, there is a pattern of localised hypermutation called kataegis in 50% of the tumours [3, 37]. These point mutations are non-recurrent, haphazard and cannot be considered as driver mutations. Further hampering the identification of driver genes is that no benign precursor of osteosarcoma is known. This is in contrast with for instance colorectal cancer, in which a benign precursor can be used to investigate multi-step progression behind tumorigenesis. Nevertheless, recent next-generation sequencing studies have revealed known and novel recurrent genetic alterations in osteosarcoma (Table 3). Most genes that were found to be altered are involved in maintaining genomic stability. Among the most commonly altered genes in osteosarcoma are the main players in maintaining genome stability: *TP53* and *RB1*.

**Table 3:** Overview of recurrent alterations found in conventional osteosarcoma

Gene	Type of alteration	Somatic/germline	Function	Frequency in sporadic OS (%)	Literature
TP53	Translocation; deletion; mutation	Germline (Li-Fraumeni syndrome) and somatic	Genome stability; cell cycle control	47–90	[3, 36–38]
RB1	Mutation; deletion	Germline (retinoblastoma) and somatic	Genome stability; cell cycle control	29–47	[3, 37, 38]
MYC	Amplification	Somatic	Cell proliferation	39	[39]
CCNE1	Amplification	Somatic	Cell cycle control	33	[39]
DLG2	Deletion	Somatic	Cell signalling	29–52	[3, 40]
COPS3	Amplification	Somatic	Signal transduction	20–39	[36, 37]
AURKB	Amplification	Somatic	Cell cycle	13	[39]
PTEN	Mutation; deletion; copy number alteration	Somatic	Cell cycle control	12–50	[36–38]
CDKN2A	Deletion	Somatic	Cell cycle control	15	[38]
ATRX	Mutation; deletion	Germline (ATR-X syndrome) and somatic	Genome stability; chromatin remodelling; ALT	10–29	[3, 33, 34, 36–38]
CDKN2A	Mutation; deletion	Somatic	Cell cycle control	10	[36]
CDK4	Amplification	Somatic	Regulates RB activity	9–11	[39, 41]
MDM2	Amplification	Somatic	Regulates P53 activity	5–12	[37, 41]
IGF1R	Mutation; amplification	Somatic	Bone growth and development	5	[36]
AKT	Amplification	Somatic	Cell proliferation; apoptosis	5	[39, 42, 43]
RECQL4	Mutation	Germline (Rothmund-Thomson syndrome)	DNA repair; genome stability	0	[32]
WRN	Mutation	Germline (Werner syndrome)	DNA repair; genome stability	0	[32]
BLM	Mutation	Germline (Bloom syndrome	DNA repair; genome stability	0	[32]

### TP53 and RB1

Mutations in *TP53* can be found in germline or can be sporadic. Previously, using immunohistochemistry or sequencing of the DNA binding domain of *TP53*, mutations were detected in only 20% of osteosarcomas [44]. Interestingly, the more sensitive whole genome sequencing studies can detect more sub clonal mutations and reveal a much higher percentage (47–90%) of osteosarcomas harbouring *TP53* alterations [3, 36–38, 45]. Furthermore, many *TP53* alterations involve structural alterations, most often consisting of translocations in the first intronic region of *TP53*, which is 10 kb in length. These alterations can only be detected with whole genome sequencing [46].

The second most frequently altered gene in osteosarcoma is *RB1* (retinoblastoma 1), involved in blocking cells from entering S phase of the cell cycle [47]. Loss of Rb function in osteosarcoma therefore leads to a loss in Rb blockade of cell division. In addition to germline mutations, somatic mutations in *RB1* were identified in 29–47% of osteosarcomas [3, 38].

The importance of *TP53* and *RB1* in osteosarcoma genesis is illustrated by the fact that patients with germline mutations in *TP53* and *RB1* are highly susceptible to cancer and frequently develop sarcomas. Different in vitro and in vivo studies confirm the important role of *TP53* and *RB1* mutations in sarcoma genesis [48, 49]. For example, homozygous deletion of *TP53* and *RB1* in osteogenic differentiated murine MSCs gives rise to osteosarcoma when injected into mice [49], while heterozygous deletion of *TP53* is sufficient to induce osteosarcoma in a mouse model [48].

### Regulators of p53 and Rb activity

*MDM2* (mouse double minute 2 homologue) regulates p53 activity by ubiquitinating p53 protein leading to proteasomal degradation of p53 [50]. Up to 12% of high-grade osteosarcomas have amplification of the *MDM2* gene at 12q13-15, but this is higher in low-grade central osteosarcoma and parosteal osteosarcoma, with around 29% and 67–79% *MDM2* amplification, respectively [41, 51] (Table 2). The *CDK4* gene (cyclin-dependent kinase 4) is located within the same region at 12q13-15 [52] and regulates Rb activity by phosphorylating Rb, resulting in deactivation of Rb. *CDK4* and *MDM2* are often co-amplified and overexpressed in osteosarcoma. *CDK4* is amplified in 67% of parosteal osteosarcomas, but rarely in high-grade osteosarcoma (9%) [41, 53]. As the percentage of *CDK4* and *MDM2* amplifications in low-grade central osteosarcoma and parosteal osteosarcoma are much higher than in high-grade osteosarcoma, most likely the *CDK4/MDM2* amplified high-grade tumours represent progression from low grade osteosarcoma [53].

Rb activity is also regulated by p16, which normally inhibits both CDK4 and CDK6. P16 is encoded by the *CDKN2A* gene at chromosome 9p21.3, that also encodes for p14. Homozygous deletion of the *CDKN2A* locus, which is associated with poor prognosis in osteosarcoma, eradicates both expression of p16^Ink4A^ and p14^ARF^, of which the latter is a negative regulator of MDM2 [38, 54–56]. Therefore, deletion of p16 and p14, similar to co-amplification of *CDK4* and *MDM2*, leads to inactivation of both the p53 and Rb pathway.

### Other genome maintenance pathways

In addition to the p53 and Rb pathway, also other pathways involved in maintaining genome stability can be affected by mutations, both in sporadic as well as hereditary osteosarcoma. For instance, *ATRX* mutations can be found both as germline or somatic mutations [57], which is in contrast to mutations in RecQ-like helicases where only germline mutations have been identified. Around 29% of osteosarcomas harbour somatic mutations in *ATRX* [3]. The role of *ATRX* mutations in osteosarcoma genesis is largely unknown. *ATRX* is involved in chromatin remodelling and plays an important role in maintenance of chromosome stability [58]. Loss-of-function mutations in *ATRX* can lead to activation of the alternative lengthening of telomeres (ALT) pathway, maintaining the length of chromosome ends [59]. ALT is found in 59% of osteosarcomas, which is much higher as compared with other cancers such as carcinomas (5–15%) [60].

DNA repair is essential in maintaining genome stability. For instance, homologous recombination, the DNA repair pathway in which BRCA plays an important role, is crucial in maintaining genome stability. A recent whole exome sequencing (WES) study revealed a subset of osteosarcomas resemble features of *BRCA* mutant tumours [38]. These tumours show loss of heterozygosity, genomic instability and a mutation signature of substitutions and deletions that is also found in breast cancers with *BRCA1/2* mutations. Around 80% of osteosarcomas show this BRCAness signature [38]. As this signature is linked to defects in homologous recombination, this vulnerability might be exploited with PARP inhibitors based on the principle of synthetic lethality. Indeed, different in vitro studies with osteosarcoma cell lines show that osteosarcoma cells are sensitive to PARP inhibitors [61, 62]. These results are promising, suggesting a possible new therapeutic strategy for osteosarcoma. However, further investigation on homologous recombination deficiency and PARP inhibitor sensitivity in osteosarcoma is needed.

### Hormonal pathways

Although the genes that play a role in genome stability are among the most frequently mutated genes in osteosarcoma (*RB1*, *TP53*, *CDK4*, *MDM2*, *ATRX*), these genes function in essential cell survival pathways. Therefore, these genes are difficult to specifically target in the treatment of osteosarcoma. Fortunately, also mutations in other genes are frequently found that are easier to target as they are involved in hormonal pathways. For example, mutations in genes involved in IGF (insulin-like growth factor) signalling, including the IGF1 receptor (*IGF1R*), were identified in around 7–14% of osteosarcomas, with many of these genes having altered activity compared with normal human osteoblasts or mesenchymal stem cells [36, 63]. The IGF signalling pathway is known to be important in normal bone growth, bone development and bone metabolism, and it is therefore not surprising that it might also play a role in osteosarcoma pathogenesis [64, 65]. These findings provide a rationale to explore anti-IGFR therapy as a treatment strategy for a subset of osteosarcomas.

The oestrogen hormonal pathway is also altered in osteosarcoma. Healthy osteoblasts normally express oestrogen receptor alpha (ERα), but this is lacking in osteosarcoma [66]. Until recently, the mechanism behind the inactivation of oestrogen receptor in osteosarcoma was not known. In a recent study, it was found that ERα was hypermethylated in osteosarcoma, which can be ameliorated by the DNA methyltransferase inhibitor DAC [67]. DAC could re-express ERα and subsequently restored defective osteogenic differentiation and inhibited proliferation in osteosarcoma cells. This study illustrates that epigenetic alterations such as hypermethylation of genes are also important in osteosarcoma genesis.

### What is driving osteosarcoma genesis?

Although recent sequencing efforts did not identify specific druggable osteosarcoma driver genes, they did reveal new and known recurrent genetic events involved in osteosarcoma that shed light on its initiation (Table 3). Most of the mutated genes function in genome maintenance pathways and the majority of osteosarcomas show genome instability in the form of chromoanagenesis [36]. Therefore, it is reasonable to assume a connection between these specific genetic mutations and chromoanagenesis, especially chromothripsis.

A main player in maintaining genome stability, *TP53*, was linked to chromothripsis in patients with Li- Fraumeni syndrome [68]. Furthermore, rats with a heterozygous deletion of *TP53* developed osteosarcomas, with chromothripsis and other complex structural rearrangements [69]. As cells with aberrant *TP53* have impaired cell cycle control [70], *TP53* loss-of-function alterations can facilitate chromothripsis by allowing cell cycle progression despite DNA damage [68]. Thus, cells with inactive *TP53* and DNA damage from chromothripsis will proliferate uncontrollably. Moreover, mutations in the DNA binding domain of TP53 can cause a neomorphic gain-of-function, that could very well contribute to the initiation of chromothripsis itself [71].

However, *TP53* alone cannot explain all tumours with chromothripsis, as is evident from studies that illustrate there are tumours with functional *TP53* with chromothripsis, and tumours with aberrant *TP53* without chromothripsis [72, 73]. Genes functionally similar to *TP53* might also be able to initiate and/or maintain chromothripsis. Whether this is the case for osteosarcomas needs to be further elucidated.

It is striking that for osteosarcoma, many different genes have been identified and with each new sequencing study, the list of potential driver genes is ever-growing. Whether a genetic alteration—in *TP53* or other genes—is a cause or consequence of chromothripsis remains unknown. One could argue whether the identified altered genes in osteosarcoma should be called “driver events” if these genetic alterations are the consequence of a single catastrophic event, such as chromothripsis. Therefore, perhaps the answer to what causes osteosarcoma could be found by discovering what causes chromothripsis. Different mechanisms have been proposed to what causes chromothripsis, such as micronuclei formation with DNA damage, telomere attrition and chromosome pulverisation by DNA damaging agents [74, 75]. Which event is the ultimate cause of osteosarcoma, is not yet known.

## Conclusion

There is an on-going shift from traditional cancer classification based solely on histopathology towards incorporation of molecular pathology in routine diagnostics, which ultimately can aid diagnostic decision making. Among the group of bone forming tumours of the skeleton, the use of deep sequencing has unravelled the molecular background of osteoid osteoma and osteoblastoma. The discovery of *FOS* and *FOSB* rearrangements found in osteoid osteoma and osteoblastoma have not only given insight in tumorigenesis, but have also provided the bone tumour pathologist with a novel diagnostic tool to improve diagnostic accuracy.

For high-grade osteosarcoma, due to its complex genomic background, no specific, recurrent genetic alteration has been found that can explain tumorigenesis, or can be used for diagnosis or treatment. Even though the number of publications on drugs that allegedly inhibit osteosarcoma growth has exponentially increased over the past few years, these claims are often based on in vitro studies including one single cell line [76]. Most of these publications are from Chinese institutes and often consist of investigations on the effect of traditional medicine on osteosarcoma. The remarkable increase of these studies is most probably the corollary of the convenient tissue culture properties of osteosarcoma cell lines and obscures findings of real significance.

Nevertheless, in the last years, several deep sequencing studies have been published that contribute towards the understanding of osteosarcoma pathogenesis. These next-generation sequencing studies have revealed underlying mechanisms, such as chromothripsis and kataegis, as well as a number of genes and pathways associated with osteosarcoma, especially those involved in genome maintenance (*TP53*, *RB1*, *ATRX* and homologous recombination) or hormonal signalling (IGF and ER signalling). The results from these studies could be the stepping stone towards the development of novel diagnostics/prognostic markers or treatment options. Since most of the alterations that were identified are not recurrent and involved in crucial processes in the cell such as genome stability, cell cycle and DNA repair, it will be a huge challenge for the coming decade to translate these findings into novel treatment options. In contrast to targeting genes involved in maintaining genome stability, such as *TP53* and *RB1*, targeting the hormonal pathways, especially IGF and oestrogen or targeting DNA repair, for example by PARP inhibition, seem more promising.
